# Re-evaluating what makes a phage unsuitable for therapy

**DOI:** 10.1038/s44259-025-00117-z

**Published:** 2025-05-29

**Authors:** Bradley W. M. Cook, Alexander P. Hynes

**Affiliations:** 1 Cytophage Technologies Inc., Oak Bluff, MB Canada; 2 Department of Biochemistry and Biomedical Sciences, McMaster University, Hamilton, ON Canada; 3 Department of Medicine, McMaster University, Hamilton, ON Canada; 4 Farncombe Family Digestive Health Research Institute, McMaster University, Hamilton, ON Canada; 5 Michael G. DeGroote Institute for Infectious Disease Research, McMaster University, Hamilton, ON Canada

**Keywords:** Antimicrobials, Bacteriophages, Pathogens, Phage biology, Policy and public health in microbiology, Virology, Phage biology, Therapeutics, Health policy

## Abstract

One of the biggest challenges in phage therapy is selecting the ‘right’ phage. What constitutes a ‘good’ phage is unclear, but regulators are converging on exclusion criteria for ‘bad’ phages. Here, we examine three commonly applied criteria: the lack of virulence/antibiotic resistance, the inability to transduce, and being strictly lytic (virulent). We assess the risk posed, the tools for determining the criteria, and the potential impact of the criteria.

## Perspective

As of 2019, nearly 1.3 million deaths worldwide were directly associated with antimicrobial resistant (AMR) infections^1^. Moreover, if AMR trends continue, some projections indicate that global mortality could reach 10 million deaths annually by 2050^2^. A crisis is looming, and has led to concerted efforts to deploy alternative antimicrobial strategies, such as the use of bacteriophage therapy. Although promising clinical outcomes have been observed in compassionate use settings over the last 100 years^3^, and recent case-series offer increasingly compelling evidence of their potential^4^, phage therapy has yet to demonstrate its efficacy in a clinical trial.

One of the key challenges of phage therapy is finding the right phage for the job. As phage host ranges are typically very narrow—often down to the strain level^5^, there is usually no ‘broad-spectrum’ phage available. While incredible strides have been made to establish libraries with thousands of sequenced phages^6^, the problem is on a completely different scale from that of selecting the right antibiotic. It is often a case of personalized medicine: in vitro efficacy can be quickly (and often cheaply) tested, but scaling up to in vivo tests for each potential phage is almost inconceivable. Accordingly, the field as a whole has had to make educated assumptions about what in silico and in vitro parameters will predict treatment success.

While there is disagreement on what makes a good (i.e. high therapeutic value) phage, the literature has been surprisingly consistent on what makes an inappropriate one: largely bioinformatics-based exclusion criteria, centered around concerns for patient safety. On either side of the Atlantic^7,8^, the consensus is that phages should carry no virulence/toxin or antimicrobial resistance genes, not be able to transduce, and be strictly lytic (virulent). Here we examine each of these criteria in-depth, discussing their justification, how they are applied, and whether they are appropriate.

## Criterion 1: the phage should not encode virulence factors or antimicrobial resistance genes

Scientists do not wish to add genetic material that could risk making an infection worse, or make it harder to treat. Typified by our understanding of the phage-encoded cholera and shigella toxin proteins (Ctx, Stx), which are some of the most important virulence factors contributing to cholera and hemolytic uremic syndrome, respectively—we have extremely clear examples of how the genetic content of a phage can drive disease in humans^9,10^. Other virulence factors, like the phage Gifsy-3 protein SspH1, facilitate persistence of the host *Salmonella* in mouse models of infection^11^.

Our ability to predict virulence is constrained by similarity to known virulence factors—such as the Virulence Factors Database^12^—which are already known to be very diverse. Accordingly, it is both challenging to apply as an exclusion criterion, and challenging to estimate the actual prevalence of virulence factors on phages. The latter is typically estimated by looking at prophage regions in bacterial genomes and determining the prevalence of phage-associated virulence factors. In pathogens, this already biases considerably for increased abundance. One study in ESKAPE pathogens found prevalences ranging from very low (0 and 1.2%) in *Acinetobacter* and *Klebsiella*, to very high (70.4% and 72.3%) in *Enterococcus cloacae* and *Staphylococcus aureus*^13^. Another study looking for the well-studied toxins in *Vibrio cholera* found that 15% of marine (presumably, non-pathogenic) isolates contain a prophage-associated toxin^14^.

In contrast, the search for antimicrobial resistance genes is better constrained. The databases of known antimicrobial resistance determinants are more robust, with tools like Resfinder^15^ and the Resistance Gene Identifier^16^. Environmental filtrates/viral fractions frequently contain antimicrobial resistance genes—especially when specific genes are targeted for selective amplification^17^. However, direct examination of phage genomes suggests that phages from human and animal fecal material collected from effluent and farm environments rarely contain antimicrobial resistance genes^18–21^, and that many of the cases where they might be predicted, are not associated with microbial resistance. In rare cases where the phages *are* associated with resistance, this was not attributed to an antimicrobial resistance gene on the phage^22^. For a direct comparison, the prior ESKAPE pathogen study found phage-associated resistance genes much more constrained than virulence factors, with prevalence ranging from 1.8% to 12.6% in all but *Klebsiella*, where it peaked at 20.9%^13^.

We believe there is strong evidence to support excluding phages with virulence factors, although there may be value in better refining which factors to prioritize. While the existing screening is restricted by our knowledge of virulence factors, it is still valuable. In contrast, the prediction of antimicrobial resistance genes in phages is more robust, but appears to poorly associate with bacterial resistance, and may be less valuable a criterion. However, in both cases the application of the criteria is quick and inexpensive, and the frequency with which a phage is discarded based on these analyses is likely very low, so little damage is done by erring on the side of caution.

## Criterion 2: the phage should not be capable of transduction

As summarized in a review article by Borodovich et al.^23^, some bacteriophages are known to contribute to horizontal gene transfer, a process which can facilitate the spread of virulence genes, antimicrobial resistance genes, and fitness determinants—even when the phage genome contains no such genes (Criterion 1). While in theory, the rapid lysis of cells and release of intracellular DNA could contribute to horizontal gene transfer through natural competence, this is likely unavoidable and cannot be a reasonable exclusion criterion. The concerns about horizontal gene transfer are usually specific to transduction—the process by which during morphogenesis, a phage packages some host genetic material into its capsid. Upon subsequent infection, that non-phage genetic material is delivered into another host cell to establish itself as part of that genome, primarily through recombination^23^.

The Transduction assay is a classical approach to determine whether a phage can in fact, transduce deliberately selected traits. A bacterial strain containing a well-defined marker (typically an antibiotic resistance or auxotrophic marker) is infected with a phage of interest, the resulting lysate is used to infect the same strain without the marker, then plated on selective medium. Only cells that have acquired the marker from the phage will grow, and are referred to as transductants^24–26^. These types of assays offer a quantifiable level of transduction, generally in the 10^−3^–10^−8^ PFU/mL range)^26–31^. While not laborious, the immense selective pressure for a targeted and well-defined marker over a short time likely greatly overestimates its impact^32,33^.

Another common analysis to detect transduction potential is simply to sequence the phage, and identify non-phage reads contained within the capsid (i.e. protected from DNAse digestion). In principle, this offers a hypersensitive and quantifiable tool that avoids the selection biases from transduction assays. Recent examples indicate that transducing phages can be readily detected in lysates^34^. However, many find disparate results during re-analysis of sequences from databases and highlight overestimations of transduction-potential: chalked-up to improper assignment of reads, bacterial contamination and, when quantitative PCR is used, amplification bias^18,19,21^. When more stringent definitions are applied, it would appear that antibiotic-resistance genes are very rarely present in transducing phages from human and animal fecal samples^18,19^. In keeping with this, a minireview of horizontal gene transfer in clinical settings attributed most movement of antibiotic resistance genes to conjugation or natural transformation, with the role of transduction “poorly characterized”^35^. The field would benefit from clear and uniform experimental procedures and data interpretations to ascribe transduction capability.

It is unsurprising that the effect of transduction does not appear to be as large in practice as is feared. Given the narrow host ranges of phages and the typical requirement for (often homologous) recombination for the acquisition of a new trait, most phages are not moving markers great genetic distances. While the target strain(s) may have diverged in the host, the risk here is one of potentially reshuffling alleles, rather than introducing ‘new’ ones. The exception is if the phage, prior to administration, is propagated on a host containing undesirable traits—something that is best avoided for multiple, critical reasons^8,36^.

Despite many guidelines claiming that phages selected for therapy should not be able to transduce, most phage therapy applications do not report these tests, and this exclusion criterion rarely appears to be applied. If it were, it is unclear whether the tools used to detect it would accurately quantify the risks—they consistently appear to inflate it. It is likely that the sequence-detection based approaches would rule out many phages that are functionally incapable of transduction. Ultimately, we believe—as do others^36^—that the risk of dissemination of fitness determinants is low, and will clearly have a far less negative impact on the patient than an untreated bacterial infection.

## Criterion 3: the phage should be strictly lytic (virulent)

This is the most consistently applied criterion for excluding a phage. While in theory, this excludes any alternate life cycles: the chronic cycle of filamentous phages and lysogenic cycle of temperate phages. In clinical practice, this is primarily applied to exclude the latter group. The commonly cited reasons to avoid these fall under three broad categories; concerns about transduction, concerns about lysogenic conversion, and regulatory/societal concerns because these may genetically alter their hosts. Without dismissing the importance of the latter—an important limitation—it falls outside our expertise, thus we will constrain ourselves to discussing the former two, scientific points.

Transduction is strongly associated with temperate phages, especially since temperate phages like P1 and P22 were commonly used for genetically-manipulating lab strains^37,38^. However, this has led to a misconception that temperate phages are better transducers than virulent phages, something unsupported by the literature^39–41^. In fact, virulent phages may tend to move markers across wider phylogenetic distances^42^. There are, however, two kinds of transduction unique to temperate phages—specialized transduction and lateral transduction, which occur either due to errors in the precision or timing of excision from the bacterial chromosome^43,44^. The former is limited to moving genes directly adjacent to the phage site of integration and therefore poses a far smaller—and easily quantifiable—risk of facilitating horizontal gene transfer, especially for genes of concern. The latter has been shown to transfer genes as far as 500 kb from the phage integration site^45^ but has only been demonstrated in phages already capable of generalized transduction; non-specific *pac*-type packaging^23^. Given that generalized transduction of a phage depends on packaging strategy rather than life cycle, and moreover that temperate-specific transduction is only a concern when generalized transduction is also a concern, we feel there is no reason to dismiss temperate phages on these grounds alone. Criteria for transduction should be assessed independently (see Criterion 2).

The second scientific concern is that of lysogenic conversion, where the infected bacterium’s phenotype is altered by the prophage. The classic example was hinted at earlier—toxin-encoding phages responsible for the pathogenicity of *Escherichia*, *Shigella*, and *Cholera* species^9,10^. Here the potential risk is very high. However, in these cases, the first exclusion criterion (genomes devoid of virulence factors or antimicrobial resistance genes) would rule out these phages, regardless of their temperate nature.

In contrast, there are examples of lysogenic conversion that would not be caught by earlier exclusion criteria. Most intriguing among these are the fitness advantages conferred by a prophage. Because a prophage’s fate is closely tied to its host, natural selection has favored temperate phages that minimize their burden to, or in fact benefit their host. Undoubtedly, benefits are inherently varied, from subtle shifts in carbon metabolism^46^ that benefit the host under starvation, to influencing dormancy (sporulation) and host colonization^47^. Bacteria can be so dependent on these benefits, that when cured of all (defective) prophage regions, *E. coli* K-12 was a far less fit than the unaltered strain^48^. As many of these condition-based, fitness altering examples appear to arise from gene regulation by the prophage, these are unpredictable, and could even be missed by targeted transcriptomics during infection-mimicking conditions.

Outweighing these subtle fitness benefits are the two universal advantages that inherently come from carrying a prophage: lysogens can deploy the phages to kill related competitors, gaining a sizeable competitive advantage. From a therapeutic standpoint, this is of no concern, as it acts to reduce the bacterial load. The second is immunity to superinfection by the same or related phages, ‘protecting’ their investment in their host. This can occur through a variety of mechanisms spanning Superinfection Immunity (repressor based), Superinfection Exclusion (Preventing DNA entry), or other phage-resistance mechanisms encoded by the phage. While any antibiotic or phage challenge will *select* for resistance, a challenge with a temperate phage also *generates* resistance—a qualitatively different problem.

In silico tools like Phage AI^49^, PhageLeads^50^ and PHASTEST^51^ can help predict phage lifestyle, primarily by using signature genes (integrases, transposases, or, with less confidence, plasmid-like origins of replication) and thus, this criterion is fairly straightforward to apply. However, simply because a phage possesses integrases or transposases does not mean it is temperate, and even if it is capable of lysogeny, does not mean it will frequently (or even ever, in any particular host) do so. To highlight this, Lauman and Dennis^52^ proposed replacing the term ‘temperate’ with ‘lysogenization-capable’, and showed how closely related *Acinetobacter* temperate phages varied widely in their frequency of lysogeny. Some did not form lysogens in specific strains, or under specific conditions^52^. Furthermore, a phage which can lysogenize, but has a very high frequency of induction would be, for therapeutic purposes, indistinguishable from a virulent phage. These complications further increase the value of a phenotypic screen^53^ rather than a bioinformatic one. Building on this, a new discovery further muddies the waters in defining temperate and lytic lifestyles; virulent phages – especially ‘jumbo’ phages—appear to be able to persist in bacterial isolates for generations^54^.

Although there are no convincing global estimates of the frequency of temperate phages, this criterion clearly excludes an enormous number of phages. The most recent estimate, surveying 13,713 bacterial genomes, predicted that 75.61% of them contained at least one functional prophage^55^—which not only highlights their abundance, but also the potential ease of isolation; any clinical bacterial collection already contains temperate phages ripe for use.

Furthermore, these phages are sorely needed; several prime candidates for phage therapy, such as *Clostridiodes difficile* infections, are stymied by the lack of virulent phages^56^—forcing scientists to consider therapeutic applications of temperate phages^57^. As temperate phages are a functional categorization and not an evolutionary one, the only barrier to their use is the ability to lysogenize their host, rather than some other common properties thereof. No story better highlights both the need and the utility of temperate phages than when the world’s largest collection of *Mycobacterium* phages (almost 10,000) only had a single virulent phage suitable for treating an *M. abscessus* infection. The team created virulent variants of two temperate phages through targeted genetic modification, which helped the patient enter recovery^58^.

In our view, while the prediction of a temperate—or at least lysogenization-capable—phages is good, the risks associated with administration are either pre-empted by earlier criteria (1 and 2), or simply an increased chance of treatment failure due to phage-driven resistance in an emerging lysogen population. This could be enough to justify excluding temperate phages, but the massive number of phages being discarded by this criterion, especially in consideration of a lack of virulent phage availability for a given genus, gives us pause. Surely being treated by a phage with a higher chance of emerging resistance is better than not being treated at all, especially in the current environment dominated by personalized one-off treatments, where the temperate phage selected is far less likely to see broad applications.

That said, to leverage temperate phages effectively, we should either decrease their frequency of lysogeny or increase their frequency of induction, so that lysogeny does not persist. Targeted gene editing of the repressor or integrase accomplishes this, but is also challenging to scale. A logical alternative approach is to leverage antibiotics which influence the lysis-lysogeny decision of temperate phages by either: promoting induction^59^ or prevention of lysogeny^60,61^. This can effectively re-sensitize clinical isolates to antibiotics they would otherwise be resistant to as previously demonstrated in an in vivo infection model—*Caenorhabditis elegans*^62^. The resultant potent synergy, termed ‘temperate-phage antibiotic synergy’ (tPAS) leads to bacterial eradication with temperate phages that would otherwise be very ineffective. In the aforementioned *C*. *elegans* work, even the pre-existing presence of a prophage in the *Pseudomonas* was sufficient to enable otherwise sub-therapeutic doses of antibiotic to rescue the worms^62^. Given the prevalence of prophages in clinical isolates, we have likely been (unknowingly) exploiting the therapeutic benefits of temperate phages with many antibiotics—for decades!

## Summary

The clinical parameters of phage therapy are still being delineated across the world, with Portugal being the latest country to adopt a regulatory framework^63^, adapted from the Belgian “magistral framework”^64^. Before these frameworks rigidify into a consensus which is difficult to challenge, we have an opportunity to (re)define what constitutes a “good” phage or a “bad” phage. Our examination of the common criteria presented herein suggests a rational approach to scrutinize these therapeutic tools while being fair, pragmatic and taking into account: virulence and antibiotic-resistance, transduction potential and, lysogenic capabilities. We conclude that the first is justifiable, but the other two—if applied dogmatically—are problematic. Ultimately as more trials report their outcomes, the field will gain invaluable evidence as to which criteria truly represent a “good” or a “bad” phage. Until then, we urge readers to not overlook the potential of inappropriately-labeled transducing and/or temperate phages.

## Data Availability

No datasets were generated or analysed during the current study.
